# The Complementarity of Amino Acids in Cooked Pulse/Cereal Blends and Effects on DIAAS

**DOI:** 10.3390/plants10101999

**Published:** 2021-09-24

**Authors:** Fei Han, Paul James Moughan, Juntao Li, Natascha Stroebinger, Shaojie Pang

**Affiliations:** 1 Academy of National Food and Strategic Reserves Administration, Beijing 100037, China; psj@ags.ac.cn; 2 Riddet Institute, Massey University, Palmerston North 4442, New Zealand; P.J.Moughan@massey.ac.nz (P.J.M.); N.Stroebinger@massey.ac.nz (N.S.); 3 State Key Laboratory of Animal Nutrition, College of Animal Science and Technology, China Agricultural University, Beijing 100193, China; lijuntao@cau.edu.cn

**Keywords:** pulse/cereal, TID, DIAAS, protein quality, complementarity, human

## Abstract

The aim was to study the complementary effect between cereals and pulses on protein quality. The values for the digestible indispensable amino acid score (DIAAS) in cooked cereals and pulses, given alone, and blends of cooked cereals and pulses, were determined. True ileal digestibility (TID) values of amino acids for adult humans were obtained. It is difficult to determine ileal amino acid digestibility in humans directly, and for this reason, the growing pig is often used to obtain such values, as a preferred animal model. Seven growing pigs fitted with a T-cannula at the terminal ileum were allotted to a 7 × 6 incomplete Latin square with seven semi-synthetic diets (cooked mung bean, adzuki bean, millet, adlay, mung bean + millet, adzuki bean + adlay, and an N-free diet) and six 7-day periods. The mean TID values for crude protein differed significantly (*p* < 0.05), with millet having the highest digestibility (89.4%) and the adzuki bean/adlay mixture having the lowest (79.5%). For lysine, adzuki bean had the highest TID (90%) and millet had the lowest (70%). For the mean of all the amino acids, there was a significant (*p* < 0.05) effect of diet, with the TID ranging from 72.4% for the adzuki bean/adlay mixture to 89.9% for the adzuki beans. For the older child, adolescent, and adult, the DIAAS (%) was 93 for mung beans, 78 for adzuki beans, 22 for millet, 16 for adlay, and 66 for mung beans + millet, and 51 for adzuki beans + adlay. For mung beans, valine was first-limiting, and the SAA for adzuki beans, while lysine was first-limiting for the other foods. Chinese traditional diets, containing both cereals and pulses, are complementary for most, but not all of the indispensable amino acids.

## 1. Introduction

It is estimated (Food and Agriculture Organization, FAO) that some 850 million people are chronically hungry [1]. In Central Africa and South Asia, 10% to 30% of children have protein malnutrition, and world-wide, some one billion people receive inadequate amounts of dietary protein [1,2,3]. There is a general consensus that the current and projected food insecurity needs to be addressed, while accounting, at the same time, for the sustainability of food production. Ensuring a supply of high-quality food proteins is central to this objective [4]. Moreover, recently it has been suggested that more dietary protein should be supplied from plant proteins [5].

Most plant proteins, however, are limiting in one or more of the dietary indispensable (essential) amino acids [6], while combinations of plant protein sources may have a better balance of amino acids than the individual foods. Their amino acid patterns may be complementary to one another, with one food providing the amino acid that is limiting in the other, and vice versa. In many societies, traditional diets contain both cereals (e.g., wheat and millet) and legumes (e.g., pulses and vegetable crops), which are complementary for most, but not all amino acids and, therefore, may meet protein requirements for adults, but not for optimal growth in children [7].

Given the importance of protein in nutrition, it is required that the protein quality of foods be determined accurately [8]. There are many methods for determining dietary protein quality, but the method currently recommended by the Food and Agriculture Organization of the United Nations is the digestible indispensable amino acid score, DIAAS [9,10]. DIAAS uses “state-of-the-art” measures for amino acid digestibility, to calculate the quantities of each indispensable amino acid in the protein that will be absorbed. The absorbed quantity of each amino acid is then expressed, relative to the required amount for that amino acid. The lowest ratio between the absorbed and required indispensable amino acids determines the DIAAS. With DIAAS, amino acid digestibility is determined at the end of the small intestine (terminal ileum), and the digestibility values are referred to as ileal digestibility. As the terminal ileal digesta contain considerable amounts of endogenous (of body origin) protein, these amino acids need to be subtracted from the total amino acid output at the distal ileum, which results in a calculation of standardized or true ileal digestibility (TID) [11,12]. An impediment, currently, to the widespread implementation of DIAAS is a paucity of true ileal amino acid digestibility data for human foods [9].

It is difficult to determine ileal amino acid digestibility in humans directly, and for this reason, the growing pig is often used to obtain such values, as a preferred animal model [13]. The process of protein digestion from the mouth to the end of the small intestine is similar in adult humans and growing pigs [13], and where direct inter-species comparisons have been made, true ileal amino acid digestibility values are in close agreement between the two species [14].

The objective of the study described here was to demonstrate the effects of amino acid complementarity on determined DIAAS values for individual pulses and cereals, and pulse/cereal mixtures. It was hypothesized that the cereal/pulse blends would have higher DIAAS values than the foods given alone.

## 2. Results

### 2.1. AA Compositions of the Six Cooked Pulses and Cereals

All pigs remained healthy throughout the experiment and readily consumed their diets. The amino acid compositions of the foods are given in Table 1.

### 2.2. Mean TID of CP and AA in the Cooked Pulses, Cereals, and Pulse Cereal Blends

The TID of CP and AA’s in the mung beans and adzuki beans have been reported previously [15], but the results are replicated here to facilitate a comparison with millet, adlay, and with the mixtures of foods. The TID of CP in millet was greater (*p* < 0.05) than that for ABA and adzuki bean, and was greater (*p* < 0.05) than the TID of CP in mung beans (Table 2). The TID values of most indispensable AA in adzuki beans were not significantly different from those in mung beans, except that the TID of phenylalanine and valine in adzuki beans were greater than in mung beans (*p* < 0.05). The TID values of most AA in millet were not significantly different from those in adlay, except that the values for reactive lysine and tryptophan in millet were greater than in adlay (*p* < 0.05). The TID values of most AA in MBM were not different from those in ABA, except that the values for methionine and tryptophan in MBM were greater than those in ABA (*p* < 0.05). The mean TID of the indispensable AA in ABA (72.2%) was the lowest among the values obtained for all the cooked materials, and the mean TID of the indispensable AA in adzuki beans (84.1%) was the greatest among the values obtained for all of the cooked materials, though the diet effect for TID of the mean IAA only tended towards statistical significance (*p* = 0.06).

### 2.3. DIAAS for the Six Cooked Pulses, Cereals, and Pulse Cereal Blends

The DIAAS values and the most limiting amino acid are given in Table 3. For the child (6 months to 3 years), the most limiting AA in cooked mung bean was threonine, in cooked adzuki bean it was the sulfur AA (methionine + cysteine), and in the other cooked materials it was lysine or reactive lysine. The DIAAS value was 76 for mung beans, 66 for adzuki beans, 19 for millet, 13 for adlay, 56 for MBM, and 43 for ABA. For older children, adolescents, and adults, the most limiting AA in cooked mung beans was valine, in cooked adzuki beans it was the sulfur AA (methionine + cysteine), and in the other cooked materials it also was lysine or reactive lysine. The DIAAS was 93 for mung beans, 78 for adzuki beans, 22 for millet, 16 for adlay, 66 for MBM, and 51 for ABA.

## 3. Discussion

The four cereals and pulses evaluated in this study are commonly produced and consumed in China. Millet (Setaria italic beauv.) is one of the most important food crops of the semi-arid tropics in Asia and Africa [16]. The annual production of millet in China is about two million tons [17,18]. Adlay (*Coix lacryma-jobi* L.) has been cultivated in China for more than 6000 years. At present, it is widely planted in China, with the annual production being about 0.2 million tons [18]. Mung bean (*Vigna radiate* L.) and adzuki bean (Vigna angularis Ohwi and Ohashi) are all mainly distributed in Asia. The annual production in China is about one million and 0.4 million tons, respectively, and China ranks first and second for global production [17,18]. In China, the traditional staple food contains both cereals and pulses, and is commonly consumed as a porridge, such as the traditional Chinese “eight treasure porridge” (about eight kinds of cereals and pulses cooked together). Millet with mung beans, and adlay with adzuki beans are among the most traditional meals in China, and are currently common meals for Chinese residents, as they are food combinations that are consistent with the ideas inherent in Chinese traditional medicine.

Humans usually consume mixed diets, which contain proteins from animal-based foods (e.g., dairy products, eggs, fish, and meat) and from plant-based foods (e.g., cereals, legumes, potatoes, cassava, vegetables, and fruit) [19]. When considered globally, the plant-to-animal food ratio is 65 (%) to 35 (%), but in China, the comparable ratio is 69 to 31 [20,21]. Plant-based foods (primarily cereals and pulses) have long been important sources of protein for humans in China and elsewhere. A growing desire for non-animal-based protein sources has led to interest in an enhanced inclusion of plant-based protein sources, such as cereals and pulses, into foods [22], and thus the importance of describing the protein quality of plant foods and their combinations.

The quality of a protein is largely determined by its amino acid composition and the digestibility of the dietary indispensable amino acids. Amino acid digestibility is most accurately determined by measures made at the end of the small intestine (ileum), rather than those based on fecal analysis, and although ileal digestibility may not be a perfect measure to determine net amino acid absorption, it has been shown to give accurate values and is considerably more accurate than amino acid digestibility determined over the total digestive tract [23]. TID values, for which endogenous gut amino acids have been corrected, are assumed to be additive [24,25,26]. The TIAAD values found in the present study, for the individual foods, were generally found to be additive in the mixture of foods, and the predicted and actually determined DIAAS values for the mixtures were in close agreement (data not shown).

The concentrations of AA in the cereals and pulses used here were within the range of published values [15,27,28,29,30,31]. AAs in cereals are mainly stored in the starchy endosperm of cereals, in the form of prolamins and glutenins [32,33,34]. Prolamins are the major storage proteins in millet, adlay, maize, sorghum, rye, barley, wheat, and most cereal grains [32,33,34]. However, unlike in most cereals, prolamins are the minor storage proteins in pulses, where most AAs are stored in legumins [32,33,34,35]. Legumins contain more lysine than prolamin [35]. In the present experiment, the TID of lysine in mung bean and adzuki bean were also higher than those in millet and adlay.

The quantity and quality of protein are both determinants of the adequacy of diets, for meeting protein requirements [6] and the amino acid requirements of the human depend upon age and physiological state [36]. The DIAAS values of the cereals and pulses determined here were within the range of published values [15,22,27,28,29,30,31,37,38]. Given that lysine was often the first-limiting amino acid, it was interesting to note the effect of the reactive lysine content on DIAAS. The pulses and adlay appear to be susceptible to lysine damage during cooking and storage.

DIAAS has been reported as providing more-accurate protein quality scores than the PDCAAS [36]. Based on the cut-off values for DIAAS, given in the report of the FAO expert consultation [39], mung beans and adzuki beans can be considered as “good” protein sources for humans (older children, adolescents, and adults), because their DIAAS values are greater than 75, but for children (6 month to 3 years), only mung bean is considered as a “good” protein source. Although, for all age groups, the DIAAS values for the cereal and pulse blends (mung bean + millet, and adzuki bean + adlay) were considerably higher than those of the individual cereals, the MBM and ABA are not deemed to be “good” protein sources.

## 4. Materials and Methods

The experiment was approved by and conducted in accordance with the animal care protocols of the Animal Welfare Committee of China Agricultural University (Beijing, China).

### 4.1. Sample Procurement and Preparation of Cooked Pulses, Cereals, and Pulse Cereal Blends

Mung beans, adzuki beans, millet and adlay were sourced for the study. Mung bean (variety name: Yulv No.1) and millet (variety name: Jingu No.21) were purchased from Shaanxi Province. Adzuki bean (variety name: Jihong No.16) and adlay (variety name: XingrenXiaobaike) were purchased from Hebei and Guizhou Province, respectively. The foods were cooked before they were fed to the pigs. The cooking mimicked the common method of cooking pulses and cereals for humans used in China. The mung bean, adzuki bean, and adlay were washed and soaked for 12 h with 25 °C water, but the millet was soaked for 30 min with water. All test materials had water added at 20% of the dry weight of the raw materials, but for the millet, water was added at double the dry weight of the raw materials. The material was cooked for 70 min using a commercially available cooker (passing 100 °C steam). All the cooked materials were dried (hot air circulation), cooled and ground through a 2 mm mesh prior to inclusion in the experimental diets.

### 4.2. Animal Study

#### 4.2.1. Diets

There were 7 diets in the experiment in all, including 6 semisynthetic corn starch-based diets and 1 N-free diet. Six semisynthetic corn starch-based diets were formulated to each contain 100 g/kg crude protein (the protein content in the final experimental diets ranged from 97.4 to 108.3 g/kg dry matter) and with each of the foods or food mixtures included as the sole protein source (Table 4). The mixture of mung bean + millet (MBM) consisted of 32% mung bean and 68% millet, and the mixture of adzuki bean + adlay (ABA) consisted of 40% adzuki bean and 60% adlay. An N-free diet (<5 g/kg dry matter) was used to determine the basal ileal endogenous N loss and endogenous amino acid [36]. Vitamins and minerals were included in all diets to meet or exceed current requirement estimates for the growing pig [40]. Titanium dioxide was included in all diets (0.3%) as an indigestible marker for calculating the ileal digestibility of amino acid [10,11].

#### 4.2.2. Animals

Seven growing pigs (castrated boars, (Duroc × Landrace) × Yorkshire; initial body weight: 34.1 ± 1.41 kg) each fitted with a T-cannula at the terminal ileum according to the method of Stein et al. [41] were used in the experiment.

#### 4.2.3. Study Design

A 7 × 6 incomplete Latin square with seven diets and six 7-day time periods was used. Seven growing pigs were allocated to seven diets and 6 time periods [37]. Each assay period lasted 7 d, and ileal digesta samples were collected on days 6 and 7. The whole trial period was 42 days. No pig received the same diet more than once during the experiment and there were, therefore, six independent replicates per treatment.

#### 4.2.4. Study Management

The growing pig was adopted as an animal model for protein digestion in the adult human. All pigs were individually housed in stainless-steel metabolism crates (1.4 m × 0.9 m × 0.7 m) with adjustable sides in an environmentally controlled metabolism room (22 °C ± 2.5 °C and 12 h of light and 12 h of dark). Humidity varied from 55% to 65% during the experiment. A feeder and a nipple drinker were installed in each pen. All pigs had ad libitum access to water [36]. After 14 d of recovery from surgery for implantation of the T-cannulas, pigs were fed the experimental diets over the 42-day period.

The experimental diets were given to the pigs at a level of 8% of metabolic body weight (BW 0.75) in 2 equal meals daily (07:30 and 16:30). The ileal digesta collection followed the protocol by Abelilla et al. [38]. After an adaptation period of 5 days, ileal digesta were collected continuously for 9 h daily (from 08:00 to 17:00) on days 6 and 7. Plastic bags were attached to the barrel of the cannulas and removed whenever they were filled with digesta and immediately stored at −20 °C to prevent bacterial degradation. At the end of the experiment, digesta samples were thawed, mixed by pig and period, sub-sampled, and lyophilized in a vacuum freeze-dryer (Tofflon Freezing Drying Systems, Minhang District, Shanghai, China).

### 4.3. Chemical Analysis

Before chemical analysis, ingredient, diet and digesta samples were ground through a 1 mm screen and mixed thoroughly. Ingredient, diet and ileal digesta samples were analyzed for amino acid (AA) (AOAC method 982.30 E (a, b, c)) [42]. For methionine and cysteine the samples were subjected to cold performic acid oxidation overnight and then hydrolyzed with 7.5 N HCl at 110 °C for 24 h before AA determination. Tryptophan was determined after hydrolyzing the sample with LiOH for 22 h at a constant temperature of 110 °C. The concentration of titanium in the diets and ileal digesta samples was determined using the method described by Short et al. [43]. The reactive lysine content was determined using the method described by Moughan and Rutherfurd [44]. All analyses were conducted in duplicate. Dry matter (DM) and crude protein (CP) were determined according to standard methods GB/T 6435-2014 [45] and GB/T 6432-2018 [46]. The accuracy and precision for all analyses met the requirements of the relevant method.

### 4.4. Data Analyses

True ileal amino acid digestibility was calculated (units are g/kg DMI) [15,27,28] as follows:

TIAAD = AIAAD + ((IAA_end_/AA_diet_) × 100),

where AIAAD is the apparent ileal digestibility of AA, IAA_end_ is the ileal endogenous losses of AA (N—free diet), AA_diet_ is the concentration of AA in the diet DM.

AIAAD (%) = 100 − ((AA_digesta_/AA_diet_) × (Ti_diet_/Ti_digesta_)) × 100,

where AA_digesta_ is the concentration of AA in the ileal digesta DM, Ti_diet_ is the concentration of Ti in the diet DM and Ti_digesta_ is the concentration of Ti in the ileal digesta DM.

DIAA reference ratios were calculated.

$$\mathrm{DIAA}\;\mathrm{reference}\;\mathrm{ratio}=\frac{\mathrm{mg}\;\mathrm{of}\;\mathrm{the}\;\mathrm{digestible}\;\mathrm{dietary}\;\mathrm{indispensable}\;\mathrm{AA}\;\mathrm{in}\;1\;g\;\mathrm{of}\;\mathrm{the}\;\mathrm{test}\;\mathrm{protein}}{\mathrm{mg}\;\mathrm{of}\;\mathrm{the}\;\mathrm{dietary}\;\mathrm{indispensable}\;\mathrm{AA}\;\mathrm{in}\;1\;g\;\mathrm{of}\;\mathrm{the}\;\mathrm{reference}\;\mathrm{protein}}$$

where the reference protein indispensable amino acid profile was the amino acid requirement pattern for the 0.5–3-year-old child, and the older child, adolescent, adult [39], respectively.

DIAAS was calculated using the following equation [36,39]:

DIAAS (%) = 100 × lowest value of the DIAA reference ratio.

### 4.5. Statistical Analysis

The data were analyzed using the Proc GLM model of SAS 9.4 statistical software. The pig was the experimental unit, and pig and period were random effects and diet was the fixed effect. Least-squares means were estimated. Significant (*p* < 0.05) differences in amino acid digestibility among diets were determined using orthogonal contrasts.

## 5. Conclusions

In summary, the blending of cereals and pulses increases the overall protein quality, as determined by DIAAS and compared to the cereals given alone. For both MBM and ADA, however, considerable scope remained for further improvement in protein quality. The novel data presented on the TID of the amino acids and DIAAS values for cereals and pulses, in a form as typically consumed in China, enrich the global database of DIAAS values [47].

## Figures and Tables

**Table 1:** Amino acid compositions of the foods (mg/g protein) (mean values ± standard errors).

	Mung Bean	Adzuki Bean	Millet	Adlay
Lys	63.2 ± 0.97	51.7 ± 0.22	15.2 ± 0.42	15.5 ± 0.17
R-Lys	55.5 ± 0.62	47.0 ± 2.66	18.6 ± 0.95	13.1 ± 0.17
His	31.4 ± 0.62	28.6 ± 0.03	24.5 ± 0.06	25.1 ± 0.63
Ile	38.7 ± 0.46	30.2 ± 0.08	40.5 ± 0.06	36.4 ± 0.04
Leu	72.0 ± 0.92	85.4 ± 0.19	188.0 ± 0.06	197.0 ± 0.55
Met	12.6 ± 0	12.1 ± 0.08	30.4 ± 0	20.9 ± 0
Cys	23.7 ± 0	14.9 ± 0.22	37.1 ± 0.06	32.8 ± 0.04
Phe	55.1 ± 0.76	30.5 ± 0.06	39.6 ± 0.06	37.0 ± 0.08
Tyr	19.9 ± 0.32	15.7 ± 0.08	15.7 ± 0	20.3 ± 0.08
Thr	30.6 ± 0.30	25.8 ± 0.06	36.3 ± 0.06	26.9 ± 0.25
Trp	9.2 ± 0.14	8.6 ± 0.47	15.7 ± 0.54	4.8 ± 0.08
Val	45.2 ± 0.43	36.0 ± 0.11	45.5 ± 0.06	46.6 ± 2.03
Asp	105.7 ± 1.35	85.4 ± 0.17	68.3 ± 0.06	65.7 ± 0.21
Ser	44.4 ± 0.65	35.2 ± 0.08	43.0 ± 0.06	40.6 ± 0.72
Arg	62.4 ± 0.89	50.1 ± 0.19	27.0 ± 0.12	37.6 ± 1.65
Glu	159.3 ± 2.06	124.9 ± 0.25	201.5 ± 0.06	229.3 ± 3.21
Gly	35.2 ± 0.43	29.8 ± 0.19	25.3 ± 0.06	23.9 ± 0.17
Ala	40.2 ± 0.54	32.1 ± 0.19	84.3 ± 0.06	91.3 ± 0.55
Sum of AAs	841.1	692.3	941.2	949.3

R-lys, reactive lysine; sum of AAs was calculated using reactive Lys rather than Lys. The results are the mean values with their standard errors of two tests.

**Table 2:** Mean TID of crude protein (%) and indispensable amino acids (%) in the cooked pulses, cereals and pulse/cereal mixtures.

	Mung Bean	Adzuki Bean	Millet	Adlay	MBM	ABA	SEM	*p*
Crude protein	81.9 ^bc^	79.6 ^c^	89.4 ^a^	85.2 ^abc^	86.6 ^ba^	79.5 ^c^	2.39	<0.05
Indispensable amino acids								
Lys	86.1 ^ab^	89.9 ^a^	70.1 ^c^	66.7 ^c^	77.2 ^bc^	73.1 ^c^	3.91	<0.05
R-Lys	87.1 ^a^	88.8 ^a^	76.4 ^a^	56.5 ^b^	89.4 ^a^	85.5 ^a^	5.43	<0.05
His	68.1 ^ab^	88.9 ^a^	51.2 ^b^	56.7 ^b^	51.6 ^b^	44.3 ^b^	9.01	<0.05
Ile	83.0 ^ab^	88.6 ^a^	84.6 ^ab^	85.7 ^ab^	79.9 ^bc^	75.8 ^c^	2.06	<0.05
Leu	90.6 ^ab^	93.9 ^a^	87.9 ^bc^	91.1 ^ab^	83.8 ^cd^	82.5 ^d^	1.47	<0.05
Met	83.1 ^b^	83.4 ^b^	90.4 ^a^	89.9 ^a^	88.6 ^a^	83.7 ^b^	1.60	<0.05
Cys	53.0 ^c^	52.9 ^c^	84.6 ^a^	82.1 ^a^	76.3 ^ab^	68.2 ^b^	3.28	<0.05
Phe	83.9 ^bcd^	89.4 ^a^	86.8 ^abc^	88.4 ^ab^	82.3 ^acd^	81.2 ^d^	1.75	<0.05
Tyr	76.8 ^abc^	85.1 ^a^	72.4 ^bcd^	80.9 ^ab^	59.1 ^d^	64.7 ^cd^	4.37	<0.05
Thr	76.9 ^ab^	86.6 ^a^	79.5 ^ab^	77.9 ^ab^	71.4 ^bc^	63.6 ^c^	3.29	<0.05
Trp	82.2 ^ab^	77.0 ^abc^	87.1 ^a^	66.2 ^c^	84.7 ^a^	69.3 ^bc^	4.02	<0.05
Val	82.8 ^b^	89.2 ^a^	82.2 ^b^	84.6 ^ab^	80.2 ^bc^	74.4 ^c^	2.20	<0.05
Mean	78.8 ^ab^	84.1 ^a^	80.2 ^a^	78.2 ^ab^	77.0 ^ab^	72.2 ^b^	2.68	0.058
Dispensable amino acids								
Asp	89.9 ^b^	93.8 ^a^	83.5 ^ab^	81.1 ^ab^	78.0 ^bc^	71.6 ^c^	2.70	<0.05
Ser	82.7 ^a^	88.4 ^a^	82.4 ^a^	83.4 ^a^	74.9 ^b^	73.0 ^b^	2.47	<0.05
Arg	88.1 ^abc^	93.1 ^a^	89.4 ^ab^	89.6 ^ab^	84.1 ^bc^	81.6 ^c^	2.22	<0.05
Glu	88.2 ^abc^	92.6 ^a^	87.5 ^bc^	91.3 ^ab^	84.1 ^cd^	81.8 ^d^	1.56	<0.05
Gly	54.6 ^b^	84.3 ^a^	74.4 ^ab^	69.4 ^ab^	66.0 ^ab^	51.1 ^b^	8.46	<0.05
Ala	73.4 ^c^	87.0 ^ab^	86.2 ^ab^	88.6 ^a^	79.9 ^bc^	74.8 ^c^	2.46	<0.05
Mean	79.5 ^bc^	89.9 ^a^	83.9 ^ab^	83.9 ^ab^	77.8 ^bc^	72.4 ^c^	2.97	<0.05

^a,b,c,d^ Mean values in a row with different superscript letters were significantly different (*p* < 0.05). True ileal digestibility (TID) values were calculated by correcting the values of apparent ileal digestibility for the basal endogenous losses. Values used for the basal endogenous losses were as follows (g/kg of DMI): Asp: 0.45; Ser: 0.27; Glu: 0.57; Gly: 0.67; His: 0.50; Arg: 0.21; Thr: 0.27; Ala: 0.42; Pro: 3.75; Cys: 0.28; Tyr: 0.12; Val: 0.28; Met: 0.08; reactive Lys: 0.22; Lys: 0.23; Ile: 0.19; Leu: 0.29; Phe: 0.16; Trp: 0.11. MBM: the mixture of mung bean + millet; ABA: the mixture of adzuki bean + adlay; R-lys, reactive lysine; mean TID of indispensable amino acids was calculated using reactive Lys rather than Lys.

**Table 3:** Digestible indispensable amino acid scores (DIAAS) for the cooked pulses, cereals and pulse/cereal mixtures.

	Mung Beans	Millet	MBM	Adzuki Beans	Adlay	ABA
	DIAA reference ratio (child (6 months to 3 years)					
His	1.07	0.63	0.72	1.27	0.71	0.59
Ile	1.00	1.07	0.99	0.83	0.98	0.79
Leu	0.99	2.50	1.64	1.21	2.72	1.76
Lys	0.95	0.19	0.56	0.82	0.18	0.43
Reactive Lys	0.85	0.25	0.56	0.73	0.13	0.45
SAA	0.85	2.18	1.55	0.67	1.69	1.11
AAA	1.18	0.88	0.96	0.78	0.94	0.75
Thr	0.76	0.93	0.77	0.72	0.67	0.54
Trp	0.89	1.61	1.25	0.78	0.37	0.55
Val	0.87	0.87	0.85	0.75	0.92	0.71
DIAAS	76	19	56	66	13	43
First limiting amino acid	Thr	Lys	Lys/R-lys	SAA	R-lys	Lys
	DIAA reference ratio (older child, adolescent, adult)					
His	1.32	0.78	0.90	1.59	0.89	0.74
Ile	1.07	1.14	1.05	0.89	1.04	0.84
Leu	1.07	2.71	1.77	1.31	2.94	1.90
Lys	1.13	0.22	0.66	0.97	0.22	0.51
R-Lys	1.01	0.30	0.66	0.87	0.16	0.54
SAA	1.00	2.56	1.82	0.78	1.99	1.30
AAA	1.49	1.11	1.21	0.99	1.20	0.95
Thr	0.94	1.15	0.95	0.89	0.84	0.67
Trp	1.12	2.07	1.61	0.98	0.48	0.70
Val	0.93	0.94	0.91	0.80	0.99	0.77
DIAAS	93	22	66	78	16	51
First limiting amino acid	Val	Lys	Lys/R-lys	SAA	R-lys	Lys

SAA, sulfur amino acids; AAA, aromatic amino acids; R-lys, reactive lysine; MBM: the mixture of mung bean and millet; ABA: the mixture of adzuki bean and adlay.

**Table 4:** Ingredient and nutrient composition of the experimental diets (g/100 g as fed basis).

Composition	Mung Bean	Adzuki Bean	Millet	Adlay	MBM	ABA	N-Free
Ingredient composition							
Cooked mung bean	38.0				18.7		
Cooked adzuki bean		41.9				19.7	
Cooked millet			78.8		40.0		
Cooked adlay				56.7		30.0	
Corn starch	41.0	37.2	0.3	22.3	20.3	29.4	78.0
Purified sucrose	10.0	10.0	10.0	10.0	10.0	10.0	10.0
Corn oil	5.0	5.0	5.0	5.0	5.0	5.0	5.0
Purified cellulose	3.0	3.0	3.0	3.0	3.0	3.0	3.0
Calcium carbonate (limestone)	0.3	0.6	0.6	0.7	0.5	0.6	0.2
Calcium monophosphate	1.6	1.2	1.2	1.2	1.4	1.2	2.3
Sodium chloride	0.3	0.3	0.3	0.3	0.3	0.3	0.3
Potassium carbonate							0.3
Magnesium oxide							0.1
Titanium dioxide	0.3	0.3	0.3	0.3	0.3	0.3	0.3
Vitamin–micromineral premix ^†^	0.5	0.5	0.5	0.5	0.5	0.5	0.5
Total	100.0	100.0	100.0	100.0	100.0	100.0	100.0
nutrient composition							
DM (%)	92.28	92.43	95.44	93.67	94.12	93.28	89.38

^†^ The vitamin–micromineral premix provided the following quantities of vitamins and micro minerals per kg of complete diet: vitamin A, 5514IU; vitamin D3, 2200IU; vitamin E, 64IU; vitamin K3, 2.2 mg; thiamin, 1.5 mg; riboflavin, 4 mg; pyridoxine, 3 mg; vitamin B12, 27.6 ug; D-pantothenic acid, 14 mg; niacin, 30 mg; folic acid, 0.7 mg; biotin, 44 ug; choline chloride, 400 mg; Cu, 100 mg as cupric sulfate pentahydrate; Fe, 100 mg as ferrous sulphate monohydrate; I, 0.3 mg as potassium iodide; Mn, 40 mg as manganese oxide; Se, 0.3 mg as sodium selenite; and Zn, 75 mg as zinc oxide. MBM: the mixture of mung bean + millet; ABA: the mixture of adzuki bean + adlay.

## Abbreviations

DIAAS	digestible indispensable amino acid score
PDCAAS	protein digestibility-corrected amino acid score
AA	amino acid
CP	crude protein
TID	true ileal digestibility
TIAAD	true ileal amino acid digestibility
AID	apparent ileal digestibility
AIAAD	apparent ileal amino acid digestibility
MBM	mung bean + millet
ABA	adzuki bean + adlay
DM	dry matter
DMI	dry matter intake
FAO	Food and Agriculture Organization of the United Nations
SAA	Sulfur amino acids
AAA	aromatic amino acids
His	histidine
Ile	isoleucine
Leu	leucine
Lys	lysine
Met	methionine
Cys	cystine
Phe	phenylalanine
Tyr	tyrosine
Thr	threonine
Trp	tryptophan
Val	valine
Ala	alanine
Asp	aspartic acid
Arg	arginine
Glu	glutamic acid
Gly	glycine
Ser	serine
